# Hypoglossal Nerve Abnormalities as Biomarkers for Central Nervous System Defects in Mouse Lines Producing Embryonically Lethal Offspring

**DOI:** 10.3389/fnana.2021.625716

**Published:** 2021-01-28

**Authors:** Lukas F. Reissig, Atieh Seyedian Moghaddam, Fabrice Prin, Robert Wilson, Antonella Galli, Catherine Tudor, Jaqueline K. White, Stefan H. Geyer, Timothy J. Mohun, Wolfgang J. Weninger

**Affiliations:** ^1^Department of Anatomy, MIC, Center for Anatomy and Cell Biology, Medical University of Vienna, Vienna, Austria; ^2^The Francis Crick Institute, London, United Kingdom; ^3^Wellcome Trust Sanger Institute, Wellcome Genome Campus, Cambridge, United Kingdom

**Keywords:** hypoglossal nerve (HGN or HN), central nervous system defects, mouse, phenotyping, gene knock out, HREM, DMDD

## Abstract

An essential step in researching human central nervous system (CNS) disorders is the search for appropriate mouse models that can be used to investigate both genetic and environmental factors underlying the etiology of such conditions. Identification of murine models relies upon detailed pre- and post-natal phenotyping since profound defects are not only the result of gross malformations but can be the result of small or subtle morphological abnormalities. The difficulties in identifying such defects are compounded by the finding that many mouse lines show quite a variable penetrance of phenotypes. As a result, without analysis of large numbers, such phenotypes are easily missed. Indeed for null mutations, around one-third have proved to be pre- or perinatally lethal, their analysis resting entirely upon phenotyping of accessible embryonic stages.To simplify the identification of potentially useful mouse mutants, we have conducted three-dimensional phenotype analysis of approximately 500 homozygous null mutant embryos, produced from targeting a variety of mouse genes and harvested at embryonic day 14.5 as part of the “Deciphering the Mechanisms of Developmental Disorders” www.dmdd.org.uk program. We have searched for anatomical features that have the potential to serve as biomarkers for CNS defects in such genetically modified lines. Our analysis identified two promising biomarker candidates. Hypoglossal nerve (HGN) abnormalities (absent, thin, and abnormal topology) and abnormal morphology or topology of head arteries are both frequently associated with the full spectrum of morphological CNS defects, ranging from exencephaly to more subtle defects such as abnormal nerve cell migration. Statistical analysis confirmed that HGN abnormalities (especially those scored absent or thin) indeed showed a significant correlation with CNS defect phenotypes. These results demonstrate that null mutant lines showing HGN abnormalities are also highly likely to produce CNS defects whose identification may be difficult as a result of morphological subtlety or low genetic penetrance.

## Introduction

Globally, about 2–7% of all newborns have congenital anomalies. Due to the multifactorial etiology, the prevalence varies greatly in different geographical regions (Eke et al., 2016; Bhide and Kar, 2018; EUROCAT, 2018). For Europe, it is reported with 256.3/10,000 births, with about 10% of all birth defects affecting the nervous system (Groen et al., 2017; EUROCAT, 2018; Morris et al., 2019). Due to technical advancements in prenatal diagnostic imaging, a large percentage of congenital anomalies, especially of the central nervous system (CNS) are diagnosed *in utero*. Since most of them will have serious consequences in postnatal life, an increasing number of such pregnancies are terminated (EUROCAT, 2018; Morris et al., 2019).

Congenital disorders of the CNS, which consists of the brain and spinal cord, can be caused by infectious diseases, environmental factors, and spontaneous or inherited mutations (Jurand, 1980; Verity et al., 2003). The key to optimizing existing diagnostic procedures and developing curative strategies for CNS defects is researching how the causative factors affect the formation, growth, and remodeling of the brain and spinal cord during early development. The most fruitful experimental approach has proved to be a careful analysis of the morphological, endocrine, and behavioral phenotypes of genetically modified model organisms, most commonly the mouse (Austin et al., 2004; Rosenthal and Brown, 2007; Guan et al., 2010; Waerzeggers et al., 2010; Desgrange et al., 2019; Ruberte et al., 2020).

Two-thirds of mouse lines with randomly induced single gene disruptions produce viable homozygous offspring. Likewise, heterozygote offspring of most lines are usually viable and reproducible. In contrast, homozygous individuals of approximately one-third of single knockout (KO) mouse lines are compromised, with a severity that causes intrauterine or perinatal death (Ayadi et al., 2012; Mohun et al., 2013). In such cases, researching the effect gene knockout has on morphogenesis, form and function rely almost exclusively on careful analysis of embryo morphology.

Phenotyping projects such as the Wellcome Trust funded “Deciphering the Mechanisms of Developmental Disorders” (DMDD) program (DMDD, 2018; Mohun et al., 2013) have specifically targeted such pre-and perinatal lethal mouse lines to expand our understanding of embryonic development, including that of the CNS. Since functional analysis is necessarily precluded in such mutants, programs such as DMDD have relied on cutting-edge three-dimensional imaging methods, producing digital volume data of whole embryos. Using standardized protocols, such data has facilitated systematic phenotype analysis and annotation using three-dimensional computer representations. The DMDD program used high-resolution episcopic microscopy (HREM) as its standard imaging procedure (Mohun and Weninger, 2012b; Weninger et al., 2014). Since HREM routinely produces data of whole embryos with voxel dimensions of approximately 3 × 3 × 3 μm^3^ and near histological quality, DMDD succeeded in identifying a very large number of previously undescribed and subtle phenotype abnormalities in mouse mutants harvested at embryonic day (E) 14.5 (Dickinson et al., 2016; Wilson et al., 2016a, b; Perez-Garcia et al., 2018; Reissig et al., 2019).

The study of mouse lines with pre- or perinatal lethal homozygous offspring is particularly challenging since approximately half of such lines produce homozygous embryos that do not survive organogenesis, dying before they reach E14.5 (Mohun et al., 2013). Even for those producing embryos that reach E14.5, survival to term is infrequent. Research on such lines is therefore costly. As a result, screening programs such as DMDD have been forced to rely on phenotyping small numbers of embryos from each null mutant line (Mohun et al., 2013).

This has proved to be a significant issue since the analysis of data obtained by DMDD clearly shows that despite the use of a common and inbred genetic background, mutant embryos almost always show both a variable spectrum of phenotypes and variable penetrance of each phenotype (Wilson et al., 2016a). As a result, low penetrant phenotypes may be missed or their significance underestimated.

One possible way to solve this dilemma would be if more commonly observed phenotypes could act as “markers” of other less penetrant or more variable phenotypes. In the case of CNS defects, this would require identification of a highly penetrant abnormality that provided a statistically reliable indicator of CNS malformations in a given KO-line that might otherwise be missed because of their low penetrance or subtle character.

The DMDD program accumulated phenotype data from over 700 embryos, obtained from 81 null mutant lines and therefore provides an opportunity to test whether such markers can be identified. Two commonly detected CNS-associated phenotypes in the DMDD study were abnormalities affecting either the hypoglossal nerve or the cranial arteries (Wilson et al., 2016a). We, therefore, set out to examine the frequency of these defects in DMDD embryos and to evaluate their utility as indicators of other lower penetrant and subtle malformations of the CNS.

## Materials and Methods

The study made use of material produced in the DMDD project (DMDD, 2018).

### Embryos

For the DMDD program, more than 240 mouse lines of the C57BL/6N background with randomly selected single gene deletions that caused pre-or perinatal death were produced at the Wellcome Sanger Institute (Mohun et al., 2013; Dickinson et al., 2016). Eighty one lines (493 embryos) produced homozygous offspring that survived until E14.5, all of which were included in this study without preselecting specific genes. Between 1 and 12 individuals per line were available.

### HREM Volume Data Generation

DMDD embryos harvested at E14.5 were imaged by high-resolution episcopic microscopy according to standard protocols (Mohun and Weninger, 2012a; Geyer et al., 2017a). Stacks of 3,000–4,000 inherently aligned digital images were produced and converted to digital volume data with isotropic voxels dimensions of 2.55 and 3.5 μm.

### Data Analysis and Statistics

All HREM volume data were visualized and analyzed using the software packages Amira^1^ and OsiriX^2^, which featured a customized plug-in for standardized data annotation (Wilson et al., 2016b) according to the mammalian phenotype (MP) ontology^3^.

Phenotype abnormalities were scored according to standardized protocols (Weninger et al., 2014), acknowledging the exact developmental stage and stage-specific phenotype peculiarities as defined by Geyer et al. (2017b,c).

The digital volume data was assessed by scrolling through the data sets section by section using the original sections as well as digital resections. Additionally, volume- and surface-rendered 3D models were used if required.

SPSS statistical software (IBM SPSS Statistics version 24; IBM Corporation, Armonk, NY, USA) and Microsoft Excel (Microsoft office professional 2016, Microsoft Corporation, Albuquerque, NM, USA) were used for data collection and statistical analysis.

Nonparametric Chi-square tests were performed to compare the relation of the hypoglossal nerve, cranial artery, and central nervous system abnormalities, using a significance level of *p* < 0.05.

## Results

### Frequency of Abnormalities Amongst Homozygous-Null Mutant Embryos

#### Hypoglossal Nerve

**Figure 1:**
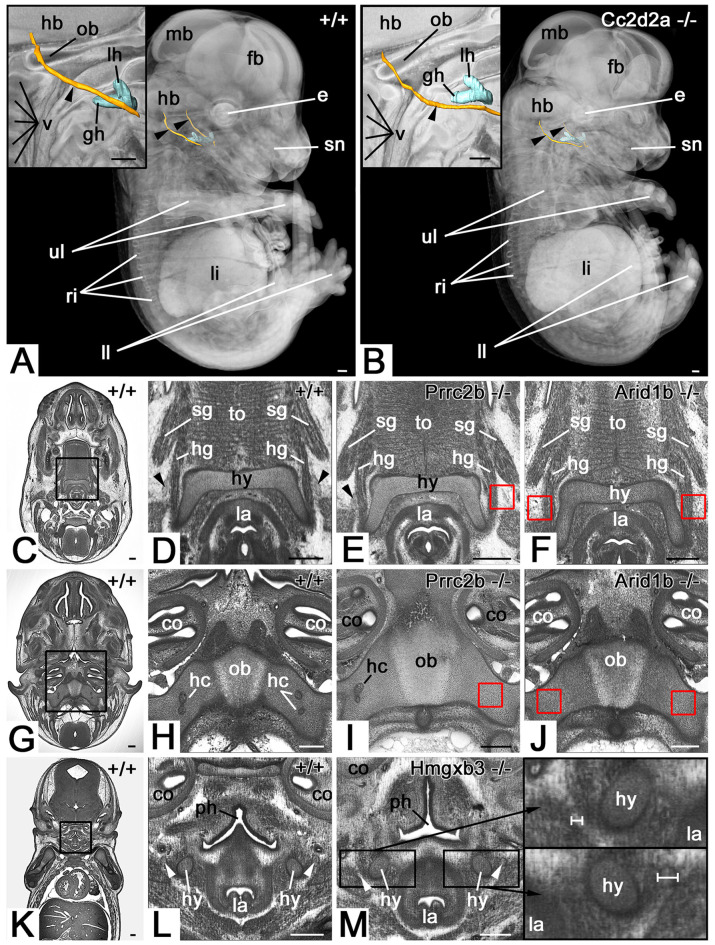
Hypoglossal nerve (HGN; arrowhead) abnormalities. **(A,B)** Abnormal topology in a *Cc2d2a* null mutant **(B)**. 3D surface models of the hyoid bone (turquoise) and hypoglossal nerve (orange) in the context of semi-transparent and median sectioned (inlay) volume models from the right. **(C–J)** The absent nerve in axial sections through HREM data of *Prrc2b* **(E,I)** and *Arid1b* **(F,J)** mutants. Red boxed areas in **(E,F)**, and **(I,J)** indicate positions at which the nerve is to be expected, but is missing. Unilateral **(E)** and bilateral **(F)** absence at the level of the hyoid bone (hy). Unilateral **(I)** and bilateral **(J)** absence at the transition through the occipital bone (ob). Note the associated absence of hypoglossal canals (hc). **(K–M)** Unilateral thinning of the nerve in coronal re-sections through HREM data of an *Hmgxb3* null mutant **(M)**. Note the different thickness (white scale bars) of the thinned nerve (top inlay) compared to the normal nerve (bottom inlay). fb, forebrain; mb, midbrain; hb, hindbrain; lh lesser horn of hyoid bone; gh, the greater horn of hyoid bone; ob, occipital bone; v, vertebra; e, eye; sn, snout; ri, ribs; li, liver; ll, lower limb; ul, upper limb; sg, styloglossus muscle; hg, hyoglossus muscle; la, larynx; to, tongue; co, cochlea; ph, pharynx; Scale bars, 250 μm.

Abnormalities of the hypoglossal nerve (Figure 1) occurred in a total of 163 (33.1%) mutant embryos (Table 1). Three types of abnormalities could be distinguished. First, the complete absence of the hypoglossal nerve was observed in a total of 75 (15.2%) mutant embryos. Second, in a total of 28 (5.7%) mutant embryos the hypoglossal nerve was thinner than in wild type embryos of the same developmental stage as judged using the staging system of Geyer et al. (2017c). Thirdly, an abnormal topology of the hypoglossal nerve was observed in a total of 81 (16.4%) mutant embryos. 330 (66.9%) mutants showed normal hypoglossal nerves on both sides.

**Table 1:** Percentage of mutant embryos and KO-lines screened in the deciphering the mechanisms of developmental disorders (DMDD) project diagnosed with abnormalities of the hypoglossal nerve (HGN), cranial arteries (CA), and central nervous system (CNS).

	DMDD KO-embryos *n* = 493	DMDD KO-lines *n* = 81	DMDD KO-lines (≥6 mutants) *n* = 47
HGN	33.1% (163)	53.1% (43)	57.4% (27)
HGN unilateral	17.4% (86)	22.2% (18)	
HGN bilateral	15.6% (77)	11.1% (9)	
HGN uni- and bilateral		19.8% (16)	
HGN absent	15.2% (75)	25.9% (21)	29.7% (14)
absent unilateral	9.1% (45)		
absent bilateral	6.1% (30)		
HGN thin	5.7% (28)	22.2% (18)	25.5% (12)
thin unilateral	3.7% (18)		
thin bilateral	2% (10)		
HGN topology	16.4% (81)	40.7% (33)	46.8% (22)
topo unilateral	9.7% (48)		
topo bilateral	6.7% (33)		
**CA**	**19.6% (97)**	**48.1% (39)**	**57.4% (27)**
**CNS**	**29.4% (145)**	**55.6% (45)**	**63.8% (30)**

*Absolute numbers in parentheses. Numbers are given for individual embryos, KO-lines, and KO-lines comprised of six or more embryos. HGN abnormalities are further subdivided by type (absent, thin, abnormal topology). On the embryo level, HGN abnormalities and subtypes are subdivided by unilateral and bilateral occurring abnormalities. On the KO-line level laterality is indicating diagnosed abnormalities in a given line being found only unilaterally, only bilaterally, or both*. Values for the main types of malformations (HGN, CA, CNS) are provided in bold font. Values for the subtypes of HGN abnormalities are provided in standard font.

#### Cranial Arteries

**Figure 2:**
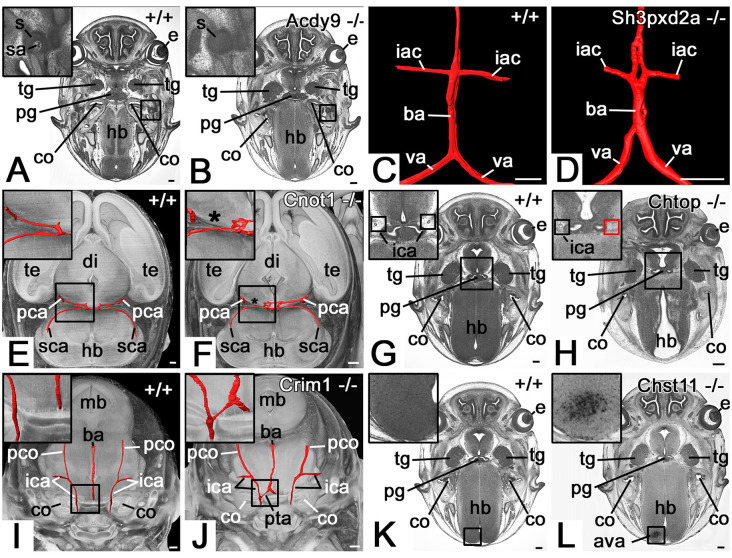
Selected abnormalities of the cranial arteries (CA). **(A,B)** Absent stapedial artery (sa) in an *Adcy9* null mutant **(B)**. Axial HREM section through head region. **(C,D)** Abnormal basilar artery morphology in a *Sh3pxd2a* null mutant **(D)**. 3D surface model (red) of the basilar artery (ba), vertebral arteries (va), and inferior anterior cerebellar arteries (iac) from dorsal. **(E,F)** The absent segment of the posterior cerebral artery (pca) in a *Cnot1* null mutant **(F)**. Surface model (red) of the posterior cerebral and superior cerebellar arteries (sca) and their connections in the context of a volume model of the head axially sectioned caudally to the models of the vessels from superior. The asterisk indicates where the missing segment is to be expected. Note the additional connection between the posterior cerebellar arteries in the mutant. **(G,H)** Absence of right-sided parasellar internal carotid artery (ica) in a *Chtop* null mutant **(H)**. Axial HREM section. Red box in **(H)** indicates the expected position of the artery. **(I,J)** Persistent trigeminal artery (pta) in a *Crim1* null mutant **(J)**. Surface model (red) of the basilar (ba) and internal carotid arteries (ica) in the context of semi-transparent, coronally sectioned volume model of the head. **(K,L)** Abnormal cerebral vessel architecture (ava) in the hindbrain (hb) of a *Chst11* null mutant **(L)**. Axial HREM section through the skull base. s, stapes; e, eye; tg, trigeminal ganglion; pg, pituitary gland; co, cochlea; hb, hindbrain, te, telencephalon; di, diencephalon; mb, midbrain; pco, posterior communicating artery; Scale bars, 250 μm.

A total of 97 (19.6%) mutants showed abnormalities of the cranial arteries (Table 1, Supplementary Table 1A). The spectrum ranged from the abnormal topology of cerebral arteries to the absence of an internal carotid artery (Figure 2).

#### Central Nervous System

**Figure 3:**
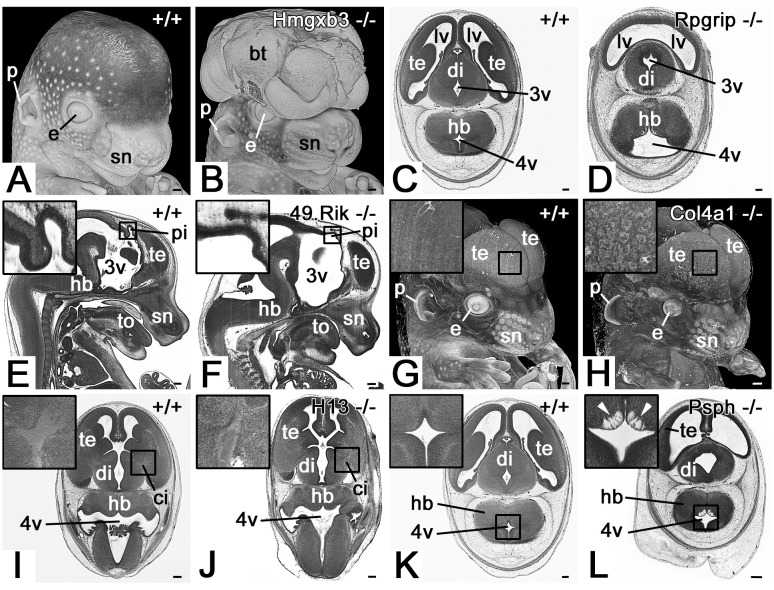
Selected large, to subtle malformations of the central nervous system (CNS), associated with hypoglossal nerve abnormalities. **(A,B)** Exencephaly in an *Hmgxb3* null mutant **(B)**. Opaque volume model of a head from ventrolateral. **(C,D)** Holoprosencephaly in a *Rpgrip* null mutant **(D)**. Axial HREM section. The lateral ventricles (lv) are in direct continuation. **(E,F)** Abnormal morphology of pineal gland vesicle (pi) in a *4933434E20Rik* null mutant **(F)**. Sagittal re-section through HREM data. Ventral to the right. **(G,H)** The abnormal architecture of the telencephalic cortex in a *Col4a1* null mutant **(H)**. Volume model from ventrolateral. **(I,J)** Abnormal internal capsule (ci) morphology in an *H13* null mutant **(J)**. Note the regular hourglass arrangement in **(I)** and the triangular arrangement in **(J)**. **(K,L)** Abnormal tissue architecture (white arrowheads) ventral to the rhomboid fossa in a *Psph* null mutant **(L)**. Axial sections through HREM data. p, pinna; e, eye; sn, snout; bt, brain tissue; 3v, 3^rd^ ventricle; 4v, 4^th^ ventricle; di, diencephalon; hb, hindbrain; te, telencephalon; to, tongue; Scale bars, 250 μm.

A total of 145 mutants (29.4%) showed abnormalities of the CNS (Table 1, Supplementary Table 1B). The spectrum ranged from very small and easy to miss subcortical tissue defects to gross malformations, such as intracerebral tumors and exencephalus (Figure 3).

### Frequency of Abnormalities Amongst Homozygous-Null Mutant Lines

We then examined the presence of hypoglossal nerve, cranial artery, and CNS abnormalities across the 81 DMDD KO-lines from which E14.5 embryos were analyzed (Table 1).

#### Hypoglossal Nerve

Abnormalities of the hypoglossal nerve occurred in 43 (53.1%) lines. The absence of the hypoglossal nerve was observed in 21 (25.9%) lines. In one of these lines (1.2%), the nerve was missing at least unilaterally in all examined mutants. In 18 (22.2%) lines at least one embryo showed a hypoglossal nerve that was thinner than in wild-types matched for precise developmental substage. However, in none of these lines was such thinning completely penetrant. In 33 (40.7%) lines at least unilateral abnormal topology of the nerve was observed. In two (2.4%) lines, this abnormality affected all examined mutants. A total of 38 (47%) KO lines yielded embryos with bilaterally normal hypoglossal nerves. In 30 (37%) lines, hypoglossal nerve abnormalities were associated with central nervous system defects.

#### Cranial Arteries

In 39 (48.1%) lines, at least one abnormality of the cranial arteries occurred. In 30 (37%) lines cranial artery abnormalities were associated with central nervous system defects.

#### Central Nervous System

In 45 (55.6%) lines at least one mutant had central nervous system abnormalities.

#### Additional Findings

Remarkably, in all eight lines in which we identified embryos with an abnormality of the developing pineal gland (*4933434E20Rik, Brd2, Chtop, Morc2a, Psph, Rpgrip1l, Sh3pxd2a, Smg9*), mutants with abnormalities of the hypoglossal nerve were present. Similarly, in all three lines in which we diagnosed abnormal cerebral cortex morphology (*4933434E20Rik, Psph, Polb*), we found mutants with either absent or thin hypoglossal nerves. In all 11 lines in which we diagnosed mutants with abnormal morphology of the internal capsule of the brain (*4933434E20Rik, Arid1b, B9d2, Cc2d2a, Dbn1, Dhx35, H13, Morc2a, Psph, Polb, Rpgrip1l*) we also found mutants with hypoglossal nerve abnormalities.

### Abnormalities in DMDD Lines With 6 or More Mutants

To identify whether hypoglossal nerve or cranial artery abnormalities have the potential to serve as indicators for abnormalities of the cranial nervous system, we checked these features in the 47 DMDD lines, which included 6 or more null mutants (Table 1, Supplementary Table 2).

#### Hypoglossal Nerve

Twenty-seven lines (57.4%) had at least one mutant embryo with uni- or bilateral abnormality of the hypoglossal nerve (absent: 14 lines, 29.7%; thin: 12 lines, 25.5%; abnormal topology: 22 lines, 46.8%). 27 lines (57.4%) had at least one mutant embryo with abnormalities of the cranial arteries. Thirty lines (63.8%) had at least one mutant embryo with a defect of the central nervous system (Table 1).

Twenty one lines (44.6%) showed hypoglossal nerve abnormalities as well as defects of the central nervous system. A chi-square test of independence proved a significant association between the presence of hypoglossal nerve abnormalities and defects of the central nervous system (*χ^2^* = 5.347, *p* = 0.021). Applying a chi-square test to the three types of hypoglossal nerve abnormalities separately, revealed no significant association for absent hypoglossal nerve (*χ^2^* = 1.87, *p* = 0.170) and thin hypoglossal nerve (*χ^2^* = 2.655, *p* = 0.103). However, there was a significant statistical association between the occurrence of either absent or thin hypoglossal nerve and central nervous system defects (*χ^2^* = 4.806, *p* = 0.028). The abnormal topology of the hypoglossal nerve was similarly associated with central nervous system defects (*χ^2^* = 5.797, *p* = 0.016).

#### Cranial Arteries

Twenty lines (42.6%) showed abnormalities of the cranial arteries as well as defects of the central nervous system. A chi-square test of independence failed to reveal a significant association between the cranial artery and central nervous system abnormalities (*χ^2^* = 2.884, *p* = 0.089).

Eighteen lines (38.2%) showed hypoglossal nerve abnormalities as well as abnormalities of the cranial arteries. A chi-square test of independence did not show any significant association between the two features either (*χ^2^* = 2.206, *p* = 0.137).

## Discussion

Systematic phenotype screens have revealed that approximately one-third of all mouse lines with a single gene disruption produce homozygous offspring which die pre- or perinatally (Mohun et al., 2013). Yet, according to data produced in the DMDD program, only about two-thirds of such lines with homozygous null embryos surviving until E14.5 have malformations of severity that sufficiently explain early prenatal death. Furthermore, about one-quarter of the DMDD lines even produce offspring that are apparently almost normal, as judged by morphological screening. However, in almost 20% of homozygous mutant embryos analyzed in the DMDD program screening, detected an absent or thin hypoglossal nerve and such defects were found in offspring from almost half the DMDD lines analyzed at E14.5. Since the hypoglossal nerve innervates the tongue and is likely to be essential for successful suckling, abnormalities such as its loss or thinning could readily explain perinatal death. DMDD data, therefore, suggest that a large proportion of homozygous mutant embryos of embryonic lethal mouse knockout lines might die because of hypoglossal nerve defects. Similarly in sub-viable lines, such defects might be responsible for the reduced number of survivors amongst pups that are otherwise largely normal.

The hypoglossal nerve is rather thin in mouse embryos and its presence, size or integrity are unlikely to be detected in routine phenotypic analysis employing lower resolution imaging methods (such as whole mount surface examination, two-dimensional histology, μMRI, or μCT imaging). Nor, to our knowledge, have previous or ongoing screens included the examination of the hypoglossal nerve in their screening protocols (Brown and Moore, 2012). We, therefore, expect that re-screening of phenotyped mouse embryos, either from existing imaging data or from a screening of additional embryos, will most likely reveal a remarkable percentage of mouse lines producing offspring with hypoglossal nerve abnormalities. If so, such findings are likely to have a significant impact on the interpretation of results from such screens.

Deciphering the genetic mechanisms underlying congenital neurological disorders and malformations is a cost- and labor-intensive endeavor and one which has proved even more challenging as a result of the variable, and often low, penetrance of phenotypes detected (Wilson et al., 2016a). For these reasons, it would be very helpful if abnormalities can be identified that are in themselves straightforward to score but are also reliably associated with lower penetrance CNS malformations. Here we have used data accumulated in the DMDD study to examine whether relatively prevalent hypoglossal nerve and head artery abnormalities can usefully act as such “biomarkers.”

To test this hypothesis, we selected all 47 DMDD lines from which annotated data from six or more homozygous mutant embryos were available at E14.5. By assessing the statistical correlation of each of these abnormalities with lower penetrance cranial CNS defects, we have found that hypoglossal nerve abnormalities can indeed act as such a biomarker.

We suggest this has two important consequences: first, it would be beneficial to re-screen mutants already analyzed in the scope of programs other than DMDD if hypoglossal nerve abnormalities have not yet been scored. If existing data is of an inadequate resolution, we would suggest the production of additional mutants and re-screening with high-resolution imaging methods. Second, for those lines showing defects in the hypoglossal nerve, detection of the associated low penetrance cranial CNS defects may well require screening of more mutant embryos.

To test this proposition, we identified those DMDD lines in which the small number of embryos initially screened were subsequently supplemented by analysis of additional samples, bringing the total to at least six embryos. From the four lines meeting these criteria, two lines (*H13* and *Cnot1*) showed hypoglossal nerve abnormalities amongst the first three mutants analyzed. None of the three in either line showed central nervous system defects. However, subsequent analysis of further mutant embryos revealed that at least one embryo from each line had defects of the central nervous system.

As the KO-lines researched in the DMDD project are mostly novel lines, previously not described in that level of detail, detailed statistics concerning penetrance of phenotypes and links to certain human diseases are not available for most of the lines. That being said, the underlying mechanisms causing HGN and CNS abnormalities and their connection in specific KO-lines were not researched. They can only be speculated about after producing further data in the scope of future studies.

DMDD analysis identified three types of hypoglossal nerve abnormalities. Of those, abnormal topology, as well as the combination of absence and abnormal thinning of the nerve, showed a statistical correlation with CNS malformations. In contrast, solitary thinning or absence of the nerve showed no comparable linkage to CNS defects. If the hypoglossal nerve is used as a biomarker, it is, therefore, essential to score both the topology and thickness of the nerve. This is not trivial and requires sufficient reference data from control embryos that have been accurately matched in the developmental stage to the mutants. Given the small size of the nerve, we have found that successful analysis requires the use of high-quality digital volume data of a resolution higher than 8 μm edge length for cubic voxels. HREM used by the DMDD program produced whole embryo data with 3 μm isometric voxel size and could therefore enable detection of very subtle tissue defects such as small cortical tissue abnormalities (as seen, for example, in Figure 3H). However, the detection of abnormal topology and absence of the HGN should also be possible using other 3D imaging modalities especially techniques based on whole specimen clearing (Spalteholz, 1914; Santi, 2011) like ultramicroscopy or optical projection tomography (Dodt et al., 2007, 2015; Becker et al., 2008; Ban et al., 2019). But measurements of the dimensions of the HGN to diagnose thinning might not be as easily possible.

## Conclusion

Our results show that approximately 20% of mutants from single knockout mouse lines that produce perinatally lethal or sub viable mutants have abnormalities of the hypoglossal nerve. Such defects are likely to cause perinatal death. Furthermore, abnormalities of the hypoglossal nerve can act as a reliable “indicator” for lower penetrance defects of the central nervous system. We conclude that screening of lines producing mutants with hypoglossal nerve defects is an efficient way to identify mouse models for CNS defects.

## Data Availability Statement

The datasets presented in this study can be found in online repositories. The names of the repository/repositories and accession number(s) can be found below: www.dmdd.org.uk website of the DMDD program.

## Author Contributions

LR, AS, SG, and WW contributed to the conception and design of the study. LR, AS, and WW contributed to writing the manuscript. FP, RW, AG, CT, JW, and TM performed data generation and collection. LR, AS, SG, and WW performed data analyses. SG, TM, and WW contributed to reviewing and editing the manuscript.

## Conflict of Interest

The authors declare that the research was conducted in the absence of any commercial or financial relationships that could be construed as a potential conflict of interest.

## Footnotes

^1^https://www.fei.com

^2^http://www.osirix-viewer.com/

^3^http://www.informatics.jax.org/vocab/mp_ontology

## References

[B1] Austin C. P. Battey J. F. Bradley A. Bucan M. Capecchi M. Collins F. S.. (2004). The knockout mouse project. Nat. Genet. 36, 921–924. 10.1038/ng0904-921 15340423 PMC2716027

[B2] Ayadi A. Birling M.-C. Bottomley J. Bussell J. Fuchs H. Fray M.. (2012). Mouse large-scale phenotyping initiatives: overview of the european mouse disease clinic (EUMODIC) and of the wellcome trust sanger institute mouse genetics project. Mamm. Genome 23, 600–610. 10.1007/s00335-012-9418-y 22961258 PMC3463797

[B3] Ban S. Cho N. H. Min E. Bae J. K. Ahn Y. Shin S.. (2019). Label-free optical projection tomography for quantitative three-dimensional anatomy of mouse embryo. J. Biophotonics 12:e201800481. 10.1002/jbio.201800481 30729697

[B4] Becker K. Jährling N. Kramer E. R. Schnorrer F. Dodt H.-U. (2008). Ultramicroscopy: 3D reconstruction of large microscopical specimens. J. Biophotonics 1, 36–42. 10.1002/jbio.200710011 19343633

[B5] Bhide P. Kar A. (2018). A national estimate of the birth prevalence of congenital anomalies in India: systematic review and meta-analysis. BMC Pediatr. 18:175. 10.1186/s12887-018-1149-0 29801440 PMC5970488

[B6] Brown S. D. M. Moore M. W. (2012). The international mouse phenotyping consortium: past and future perspectives on mouse phenotyping. Mamm. Genome 23, 632–640. 10.1007/s00335-012-9427-x 22940749 PMC3774932

[B7] Desgrange A. Lokmer J. Marchiol C. Houyel L. Meilhac S. M. (2019). Standardised imaging pipeline for phenotyping mouse laterality defects and associated heart malformations, at multiple scales and multiple stages. Dis. Model. Mech. 12:dmm038356. 10.1242/dmm.038356 31208960 PMC6679386

[B8] Dickinson M. E. Flenniken A. M. Ji X. Teboul L. Wong M. D. White J. K.. (2016). High-throughput discovery of novel developmental phenotypes. Nature 537, 508–514. 10.1038/nature19356 27626380 PMC5295821

[B9] DMDD (2018). DMDD—Deciphering the Mechanisms of Developmental Disorders. Available online at: https://dmdd.org.uk/. Accessed June 23, 2020.

[B10] Dodt H.-U. Leischner U. Schierloh A. Jährling N. Mauch C. P. Deininger K.. (2007). Ultramicroscopy: three-dimensional visualization of neuronal networks in the whole mouse brain. Nat. Methods 4, 331–336. 10.1038/nmeth1036 17384643

[B11] Dodt H.-U. Saghafi S. Becker K. Jährling N. Hahn C. Pende M.. (2015). Ultramicroscopy: development and outlook. Neurophotonics 2:041407. 10.1117/1.NPh.2.4.041407 26730396 PMC4696521

[B12] Eke C. B. Uche E. O. Chinawa J. M. Obi I. E. Obu H. A. Ibekwe R. C. (2016). Epidemiology of congenital anomalies of the central nervous system in children in enugu, nigeria: a retrospective study. Ann. Afr. Med. 15, 126–132. 10.4103/1596-3519.188892 27549417 PMC5402814

[B13] EUROCAT (2018). European Platform on Rare Disease Registration (EUROCAT). Available online at: https://eu-rd-platform.jrc.ec.europa.eu/eurocat/eurocat-data/prevalence_en. Accessed June 23, 2020.

[B14] Geyer S. H. Maurer-Gesek B. Reissig L. F. Weninger W. J. (2017a). High-resolution episcopic microscopy (HREM)—simple and robust protocols for processing and visualizing organic materials. J. Vis. Exp. 125:56071. 10.3791/56071 28715372 PMC5609318

[B15] Geyer S. H. Reissig L. F. Hüsemann M. Höfle C. Wilson R. Prin F.. (2017b). Morphology, topology and dimensions of the heart and arteries of genetically normal and mutant mouse embryos at stages S21–S23. J. Anat. 231, 600–614. 10.1111/joa.12663 28776665 PMC5603791

[B16] Geyer S. H. Reissig L. Rose J. Wilson R. Prin F. Szumska D.. (2017c). A staging system for correct phenotype interpretation of mouse embryos harvested on embryonic day 14 (E14.5). J. Anat. 230, 710–719. 10.1111/joa.12590 28185240 PMC5382591

[B17] Groen H. Bouman K. Pierini A. Rankin J. Rissmann A. Haeusler M.. (2017). Stillbirth and neonatal mortality in pregnancies complicated by major congenital anomalies: findings from a large european cohort. Prenat. Diagn. 37, 1100–1111. 10.1002/pd.5148 28837248

[B18] Guan C. Ye C. Yang X. Gao J. (2010). A review of current large-scale mouse knockout efforts. Genesis 48, 73–85. 10.1002/dvg.20594 20095055

[B19] Jurand A. (1980). Malformations of the central nervous system induced by neurotropic drugs in mouse embryos. Dev. Growth Differ. 22, 61–78. 10.1111/j.1440-169X.1980.00061.x 37281503

[B22] Mohun T. Adams D. J. Baldock R. Bhattacharya S. Copp A. J. Hemberger M.. (2013). Deciphering the mechanisms of developmental disorders (DMDD): a new programme for phenotyping embryonic lethal mice. Dis. Model. Mech. 6, 562–566. 10.1242/dmm.011957 23519034 PMC3634640

[B20] Mohun T. J. Weninger W. J. (2012a). Embedding embryos for high-resolution episcopic microscopy (HREM). Cold Spring Harb. Protoc. 2012, 678–680. 10.1101/pdb.prot069583 22661437

[B21] Mohun T. J. Weninger W. J. (2012b). Episcopic three-dimensional imaging of embryos. Cold Spring Harb. Protoc. 2012, 641–646. 10.1101/pdb.top069567 22661435

[B23] Morris J. K. Wellesley D. G. Barisic I. Addor M.-C. Bergman J. E. H. Braz P.. (2019). Epidemiology of congenital cerebral anomalies in europe: a multicentre, population-based EUROCAT study. Arch. Dis. Child. 104, 1181–1187. 10.1136/archdischild-2018-316733 31243007

[B24] Perez-Garcia V. Fineberg E. Wilson R. Murray A. Mazzeo C. I. Tudor C.. (2018). Placentation defects are highly prevalent in embryonic lethal mouse mutants. Nature 555, 463–468. 10.1038/nature26002 29539633 PMC5866719

[B25] Reissig L. F. Herdina A. N. Rose J. Maurer-Gesek B. Lane J. L. Prin F.. (2019). The *Col4a2^em1(IMPC)Wtsi^* mouse line: lessons from the deciphering the mechanisms of developmental disorders program. Biol. Open 8:bio042895. 10.1242/bio.042895 31331924 PMC6737985

[B26] Rosenthal N. Brown S. (2007). The mouse ascending: perspectives for human-disease models. Nat. Cell Biol. 9, 993–999. 10.1038/ncb437 17762889

[B27] Ruberte J. Schofield P. N. Brakebusch C. Vogel P. Herault Y. Gracia G.. (2020). PATHBIO: an international training program for precision mouse phenotyping. Mamm. Genome 31, 49–53. 10.1007/s00335-020-09829-1 32088735

[B28] Santi P. A. (2011). Light sheet fluorescence microscopy: a review. J. Histochem. Cytochem. 59, 129–138. 10.1369/0022155410394857 21339178 PMC3201139

[B29] Spalteholz W. (1914). Über das Durchsichtigmachen von Menschlichen und Tierischen Päparaten. Leipzig, Germany: S. Hierzel.

[B30] Verity C. Firth H. ffrench-Constant C. (2003). Congenital abnormalities of the central nervous system. J. Neurol. Neurosurg. Psychiatry 74, i3–i8. 10.1136/jnnp.74.suppl_1.i3 12611928 PMC1765611

[B31] Waerzeggers Y. Monfared P. Viel T. Winkeler A. Jacobs A. H. (2010). Mouse models in neurological disorders: applications of non-invasive imaging. Biochim. Biophys. Acta 1802, 819–839. 10.1016/j.bbadis.2010.04.009 20471478

[B32] Weninger W. J. Geyer S. H. Martineau A. Galli A. Adams D. J. Wilson R.. (2014). Phenotyping structural abnormalities in mouse embryos using high-resolution episcopic microscopy. Dis. Model. Mech. 7, 1143–1152. 10.1242/dmm.016337 25256713 PMC4174525

[B33] Wilson R. Geyer S. H. Reissig L. Rose J. Szumska D. Hardman E.. (2016a). Highly variable penetrance of abnormal phenotypes in embryonic lethal knockout mice. Wellcome Open Res. 1:1. 10.12688/wellcomeopenres.9899.2 27996060 PMC5159622

[B34] Wilson R. McGuire C. Mohun T. DMDD Project. (2016b). Deciphering the mechanisms of developmental disorders: phenotype analysis of embryos from mutant mouse lines. Nucleic Acids Res. 44, D855–D861. 10.1093/nar/gkv1138 26519470 PMC4702824

