# Supplementary material for: Hypoglossal Nerve Abnormalities as Biomarkers for Central Nervous System Defects in Mouse Lines Producing Embryonically Lethal Offspring
Source: Front Neuroanat. 2021 Jan 28;15:625716. doi: 10.3389/fnana.2021.625716 (PMC7876247; doi:10.3389/fnana.2021.625716)
Supplement: Supplementary file 1 [file Table_1.DOCX]

Table 1A. CNS abnormalities diagnosed in DMDD embryos. The Mammalian Phenotype Ontology terms (MP terms) grouped by portion of the brain.

| **Brain divisions** | **MP ID** | **MP term** |
| --- | --- | --- |
| **Forebrain** | MP:0000823 | abnormal lateral ventricle |
|  | MP:0000825 | dilated lateral ventricle |
|  | MP:0000826 | abnormal 3rd ventricle morphology |
|  | MP:0008536 | enlarged 3rd ventricle |
|  | MP:0011380 | enlarged brain ventricles |
|  | MP:0000632 | abnormal pineal gland morphology |
|  | MP:0000633 | abnormal pituitary gland |
|  | MP:0005315 | absent pituitary gland |
|  | MP:0004164 | abnormality in neurohypophysis morphology |
|  | MP:0013926 | absent neurohypophysis |
|  | MP:0000783 | abnormal forebrain morphology |
|  | MP:0000787 | abnormal telencephalon morphology |
|  | MP:0000788 | abnormal cerebral cortex morphology |
|  | MP:0000820 | abnormal choroid plexus morphology |
|  | MP:0008128 | abnormal brain internal capsule morphology |
|  | MP:0013850 | absent posterior commissure |
|  | MP:0001916 | intracerebral hemorrhage |
|  | MP:0002199 | abnormal brain commissure morphology |
|  | MP:0005157 | holoprosencephaly |
|  | MP:0005236 | abnormal olfactory nerve morphology |
|  | DMDDCV | abnormal forebrain tissue architecture |
|  | MP:0006254 | thin cerebral cortex |
|  | MP:0008026 | abnormal brain white matter morphology |
|  | MP:0009770 | abnormal optic chiasm morphology |
|  | MP:0009771 | absent optic chiasm |
|  | MP: 0004269 | abnormal optic cup morphology |
|  | MP: 0013820 | absent optic cup |
|  | MP: 0004268 | abnormal optic stalk morphology |
|  | MP:0001330 | abnormal optic nerve morphology |
| **Midbrain** | MP:0000897 | abnormal midbrain morphology |
|  | MP:0005277 | abnormal brainstem morphology |
| **Hindbrain** | MP:0006054 | spinal hemorrhage |
|  | MP:0000828 | abnormal 4t ventricle morphology |
|  | MP:0000841 | abnormal hindbrain morphology |
|  | MP:0000846 | abnormal medulla oblongata morphology |
|  | MP:0000955 | abnormal spinal cord morphology |
|  | MP:0000846 | Abnormal medulla oblongata morphology |
|  | MP:0008534 | enlarged fourth ventricle |
| **Whole brain** | MP:0000914 | exencephaly |
|  | MP:0001890 | anencephaly |

Table 1B. Cranial Artery (CA) malformations diagnosed in DMDD embryos.

| **MP ID** | **MP term** |
| --- | --- |
| MP:0013997 | abnormal internal carotid artery topology |
| DMDDCV | additional labyrinthine artery |
| DMDDCV | abnormal basilar artery formation |
| DMDDCV | abnormal anterior cerebral artery topology |
| DMDDCV | absent segment of superior cerebellar artery |
| MP:0004665 | abnormal stapedial artery morphology |
| MP:0010530 | cerebral Arteriovenous malformation |
| MP:0013994 | abnormal parasellar internal carotid morphology |
| MP:0014004 | absent segment of basilar artery |
| MP:0004666 | absent stapedial artery |
| MP:0004950 | abnormal brain vasculature morphology |
| MP:0013186 | abnormal basilar artery morphology |
| MP:0013822 | abnormal anterior cerebral artery morphology |
| MP:0013823 | absent segment of anterior cerebral artery |
| MP:0013840 | absent segment of posterior cerebral artery |
| MP:0013871 | abnormal stapedial artery topology |
| MP:0013992 | persistent dorsal ophthalmic artery |
| MP:0013999 | absent parasellar internal carotid artery |
| MP:0014000 | anastomosis between internal carotid artery and basilar artery |
| MP:0014006 | absent posterior communicating artery |
| MP:0020500 | persistent trigeminal artery |

Table 2. List of genes with more than 6 embryos per line which are presenting HGN, CNS and CA malformations. Gene symbol is listed for each line studied along with the number of homozygous mutant embryos analyzed and number of malformed embryos in each line.

| Gene | E14.5 homozygous mutant embryos analyzed | HGN  (No of affected embryos) | CNS  (No of affected embryos) | CA  (No of affected embryos) |
| --- | --- | --- | --- | --- |
| *1700007K13RIK<tm2b(EUCOM)* | 7 | 0 | 0 | 0 |
| *1700067k01rik* | 8 | 0 | 1 | 1 |
| *4933434E20Rik<tm1a(EUCOMM)Wtsi>* | 6 | 6 | 5 | 3 |
| *Actn4<tm1a(EUCOMM)Wtsi>* | 9 | 9 | 7 | 1 |
| *Adamts3<tm1b(KOMP)Wtsi>(MUCB)* | 8 | 0 | 4 | 5 |
| *Adcy9<tm1b(EUCOMM)Wtsi>   (MUFE)* | 9 | 0 | 0 | 1 |
| *Arid1b<tm1b(EUCOMM)HGMU>   (PMIX)* | 7 | 6 | 2 | 0 |
| *Capza2<tm1b(KOMP)Wtsi>   (MUFS)* | 9 | 7 | 0 | 0 |
| *Cbx6<tm1a(EUCOMM)Wtsi>* | 7 | 1 | 0 | 0 |
| *Cc2d2a<tm1a(EUCOMM)Wtsi>   (MCTS)* | 7 | 5 | 5 | 2 |
| *Celf4<tm1a(EUCOMM)Wtsi>   (MGBQ)* | 6 | 0 | 0 | 1 |
| *Chst11<tm1a(KOMP)Wtsi>   (MFZV)* | 10 | 0 | 0 | 1 |
| *Cmip<tm1a(EUCOMM)Wtsi>   (MEWD)* | 10 | 3 | 0 | 2 |
| *Cnot1<R535C>   (CNOT)* | 6 | 5 | 3 | 5 |
| *Cog6<tm1a(EUCOMM)Wtsi>   (MGBW)* | 7 | 1 | 1 | 0 |
| *Coro1c<tm1a(KOMP)Wtsi>   (MDWY)* | 11 | 0 | 4 | 0 |
| *Cpt2<tm1b(KOMP)Wtsi>   (MUDF)* | 6 | 0 | 0 | 0 |
| *Crim1<em1(IMPC)Wtsi>* | 10 | 1 | 0 | 5 |
| *Cyfip2<tm1a(EUCOMM)Wtsi>   (MEPG)* | 6 | 0 | 1 | 0 |
| *Ehbp1l1<tm1a(EUCOMM)Wtsi>   (MFCC)* | 6 | 0 | 1 | 0 |
| *Fut8<em1Wtsi>   (DABG)* | 6 | 2 | 1 | 1 |
| *Gas2l2<tm1a(KOMP)Wtsi>   (MGNF)* | 8 | 0 | 1 | 1 |
| *H13<tm1b(KOMP)Wtsi>   (MUFG)* | 11 | 9 | 9 | 5 |
| *Hmgxb3<tm1a(EUCOMM)Wtsi>   (MFEZ)* | 6 | 3 | 3 | 0 |
| *Morc2a<em1(IMPC)Wtsi>* | 11 | 2 | 3 | 2 |
| *Mybphl<tm1b(KOMP)Wtsi>   (MUAF)* | 6 | 0 | 2 | 2 |
| *Nsun2<tm1a(EUCOMM)Wtsi>   (MBKW)* | 6 | 2 | 1 | 4 |
| *Oaz1<tm2e(EUCOMM)Wtsi>* | 12 | 12 | 3 | 1 |
| *Pdzk1<tm2b(EUCOMM)Wtsi>   (PMCH)* | 9 | 0 | 0 | 0 |
| *Polb<tm1a(KOMP)Wtsi>   (MGLG)* | 9 | 7 | 7 | 3 |
| *Prmt7<tm1a(EUCOMM)Wtsi>   (MBVJ)* | 6 | 4 | 0 | 0 |
| *Prrc2b<tm1a(EUCOMM)Wtsi>   (MDZU)* | 9 | 8 | 2 | 1 |
| *Psph<tm1a(EUCOMM)Hmgu>   (MGJF)* | 8 | 3 | 6 | 6 |
| *Pthlh<tm1a(KOMP)Wtsi>   (MFMW)* | 6 | 0 | 0 | 1 |
| *Rala<tm1a(EUCOMM)Wtsi>   (MEAH)* | 8 | 0 | 8 | 1 |
| *Sh3pxd2a<tm1b(EUCOMM)Wtsi>   (PMAD)* | 11 | 10 | 5 | 11 |
| *Slc20a2<tm1a(EUCOMM)Wtsi>   (MEQX)* | 11 | 1 | 1 | 0 |
| *Slc25a20<tm1a(EUCOMM)Wtsi>   (MEQX)* | 6 | 0 | 0 | 1 |
| *Slc5a7* | 7 | 0 | 0 | 0 |
| *Smg9<tm1b(EUCOMM)Wtsi>   (PMDC)* | 6 | 1 | 5 | 4 |
| *Ssr2<tm1b(EUCOMM)Wtsi>   (MUHH)* | 11 | 11 | 11 | 1 |
| *Syt1<tm1a(EUCOMM)Wtsi>   (MDNJ)* | 7 | 0 | 0 | 0 |
| *Traf6<tm2a(EUCOMM)Wtsi>* | 9 | 1 | 3 | 4 |
| *Trappc9<tm1a(EUCOMM)Wtsi>   (MFWT)* | 7 | 0 | 1 | 0 |
| *Trim45<tm1a(KOMP)Wtsi>   (MBDP)* | 6 | 0 | 0 | 0 |
| *Xpnpep1<tm1a(KOMP)Wtsi>* | 6 | 2 | 0 | 0 |
| *Zmynd11<em1(IMPC)Wtsi>   (DAEA)* | 11 | 1 | 1 | 0 |
